# A mesoporous Zr-based metal–organic framework driven by the assembly of an octatopic linker†

**DOI:** 10.1039/d3cc01831h

**Published:** 2023-05-25

**Authors:** Borja Ortín-Rubio, Cristina Perona-Bermejo, José A. Suárez del Pino, Francisco J. Carmona, Felipe Gándara, Jorge A. R. Navarro, Judith Juanhuix, Inhar Imaz, Daniel Maspoch

**Affiliations:** a Catalan Institute of Nanoscience and Nanotechnology (ICN2), CSIC and The Barcelona Institute of Science and Technology, Campus UAB Bellaterra Barcelona 08193 Spain inhar.imaz@icn2.cat daniel.maspoch@icn2.cat; b Departament de Química, Facultat de Ciències, Universitat Autònoma de Barcelona Bellaterra 08193 Spain; c Departamento de Química Inorgánica, Universidad de Granada Av. Fuentenueva S/N Granada 18071 Spain; d Materials Science Institute of Madrid (ICMM), Consejo Superior de Investigaciones Científicas (CSIC) Calle Sor Juana Inés de la Cruz, 3 Madrid 28049 Spain; e ALBA Synchrotron Cerdanyola del Vallès Barcelona 08290 Spain; f ICREA Pg. Lluís Companys 23 Barcelona 08010 Spain

## Abstract

Metal–organic frameworks (MOFs) based on high-connected nets are generally very attractive due to their combined robustness and porosity. Here, we describe the synthesis of BCN-348, a new high-connected Zr-MOF built from an 8-connected (8-c) cubic Zr-oxocluster and an 8-c organic linker. BCN-348 contains a minimal edge-transitive 3,4,8-c eps net, and combines mesoporosity with thermal and hydrolytic stability. Encouraging results from preliminary studies on its use as a catalyst for hydrolysis of a nerve-agent simulant suggest its potential as an agent for detoxification of chemical weapons and other pernicious compounds.

## Abstract 2

We present BCN-348, a new high-connected MOF built from an 8-c cubic Zr-oxocluster and an 8-c organic linker. BCN-348 shows a 3,4,8-c eps net, mesoporosity, hydrolytic stability, and acts as a catalyst for hydrolysis of a nerve-agent simulant.

## Graphical abstract

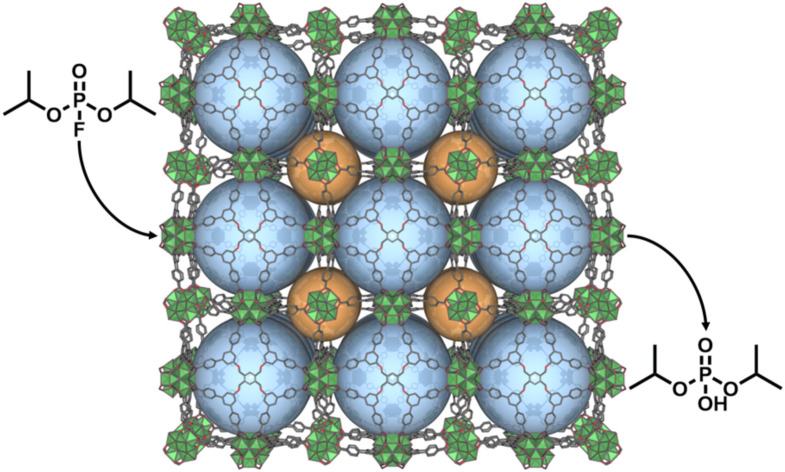

Being able to control the connectivity in reticular materials is crucial for researchers to able to design and predict the final structures and hone the properties of these materials.^1^ A current goal is the generation of highly connected metal–organic frameworks (MOFs), meaning those with a connectivity ≥ 8.^2^ This is mainly due to the tendency of such MOFs to exhibit higher chemical, thermal and compositional stability and interesting topologic profiles: for example, compared to other MOFs, they can incorporate more defects without suffering structural collapse, and their topologies are more constrained and thus, easier to predict. To date, the most common strategies to synthesise high-connected MOFs rely on the use of dendritic organic linkers to connect low-connected clusters;^3^ the use of high-connected clusters (*e.g.* 12-connected (12-c) Zr-oxoclusters^4^ and their isoreticular rare-earth (RE) analogues)^5^ or high-connected cages that are linked by low-connected linkers;^6^ and/or the simultaneous use of high-connected linkers and high-connected clusters/cages. However, even though the last of these strategies should lead to high-connected nets, only a few MOFs have actually been assembled that way. Indeed, upon searching the Cambridge Crystallographic Data Centre (CCDC) database (ConQuest v. 2.0.0, CCDC, Cambridge, UK),^7^ we were surprised to find only three high-connected MOFs: (i) one assembled by the linkage of rare trinuclear Ni(ii) clusters in a cubic conformation by an octatopic linker, giving an eps net;^8^ and (ii) two assembled by connecting 12-c nonanuclear RE clusters in a hexagonal prism conformation through an octatopic linker, affording a kce net (Fig. 1), and through a dodecatopic linker, giving a kex net.^9^

**Fig. 1:**
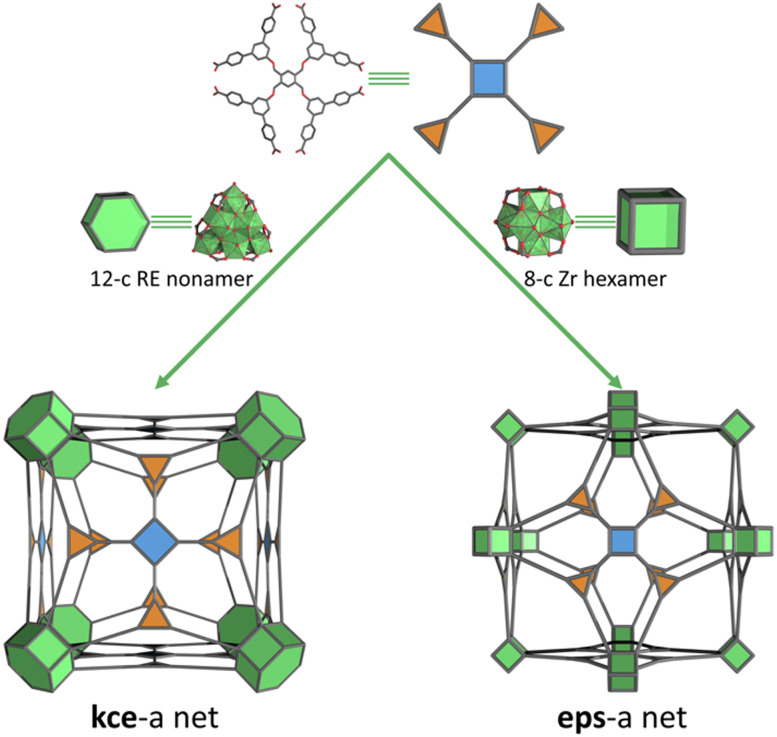
Schematic of the influence of octatopic TBCPB linker symmetry on the formation of the minimal edge-transitive kce net, when a RE hydroxo/fluorinated cluster is used (left),^9^ and of the minimal edge-transitive eps net, when a Zr oxo/hydroxo-cluster is used (right).

Another unexpected observation was the absence of MOFs assembled from (the archetypical) Zr-oxocluster and high-connected linkers.^10^ To date, Zr-MOFs have been extensively assembled from ditopic linkers (12-c cuboctahedron fcu,^4^ 8-c cube bcu^11^ and reo,^12^ 8-c hexagonal bipyramid hex,^13^ 6-c octahedron pcu^14^); triangular tritopic linkers (8-c cube the,^15^ 6-c octahedron spn,^16^ 6-c hexagonal kgd^17^); square tetratopic linkers (12-c cuboctahedron ftw,^18^ 12-c hexagonal prism shp,^19^ 8-c cube csq,^18,20^sqc^21^ and scu,^22^ 6-c octahedron soc,^23^ 6-c triangular prism stp,^24^ 6-c hexagonal she,^25^ 4-c square lvt);^26^ tetrahedral tetratopic linkers (12-c icosahedral ith,^16^ 8-c cube flu,^16^ 6-c hexagonal gar);^27^ hexagonal hexatopic linkers (6-c hexagonal hxg);^28^ and trigonal prism hexatopic linkers (12-c hexagonal prism alb).^29^

Herein we report the design, synthesis and functional validation of first Zr-MOF (hereafter called BCN-348; BCN stands for Barcelona Material), which was assembled by combining 8-c cubic Zr-oxoclusters with an 8-c octagonal linker, affording the novel 3,4,8-c eps net (Fig. 1). In this framework, the octatopic linker promotes the formation of cuboctahedral pores oriented in the six square faces, resulting in a new face-sharing, Archimedean, solid-stacking.^30^ BCN-348 is robust and exhibits remarkable porosity, as shown by the apparent Brunauer-Emmett-Teller (BET) surface area of 2564 m^2^ g^−1^ and the presence of mesopores. We exploited the exposed nature of the metal sites in the Zr-cluster and the stability of this BCN-348 in aqueous media, by testing the catalytic degradation of a nerve agent simulant assisted by the Lewis-acidic Zr sites in the absence of an amine-based buffer.

To prepare BCN-348, we combined ZrOCl_2_·8H_2_O and 1,2,4,5-tetrakis[3,5-bis(4-carboxyphenyl)phenoxymethyl]benzene (H_8_TBCPB) in the presence of trifluoroacetic acid (TFA) and *N*,*N*-dimethylformamide (DMF) under solvothermal conditions, which yielded colourless cubic crystals. Single-crystal X-ray diffraction (SCXRD) revealed an eps net, where the Zr-oxocluster exhibits an 8-connected cubic shape with four missing positions induced by the symmetry of the linker (Fig. 1). BCN-348 was solved in the cubic *Fm*3̄ space group, with a unit cell parameter of 59.677 Å, resulting in a large cell volume of 212530 Å^3^. The resulting framework presents two types of cages: a cuboctahedral cage with a diameter of 38.6 Å, which contains hexagonal (17.6 Å) and rhombic (12.1 Å) windows; and a smaller, octahedral cage with a diameter of 26.3 Å, which contains the same hexagonal (17.6 Å) windows (Fig. 2). In this structure, the octatopic ligand occupies the square faces of the cuboctahedral cages, which connect four different clusters, whereas its branches occupy the edges of the octahedral cage, which connects two clusters. Interestingly, this structure can also be described as the stacking of face-sharing Archimedean solids: in this case, cuboctahedra (Fig. 2d).^30^

**Fig. 2:**
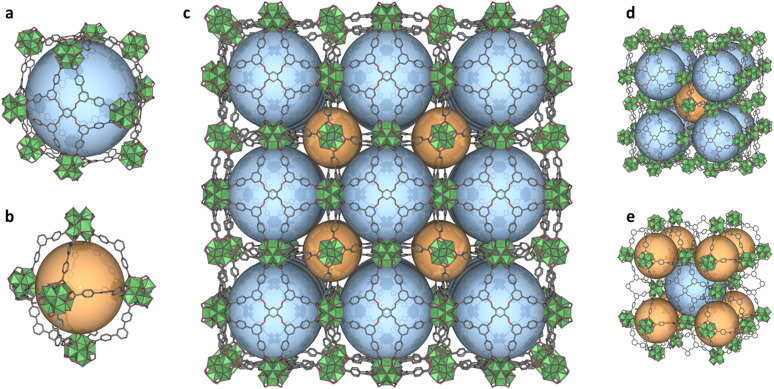
Structural illustrations of BCN-348, highlighting (a) the cuboctahedral cage assembled with 6 organic moieties located in the square faces; (b) the octahedral cage assembled with 12 partial organic moieties located at the edges; (c) the overall structure of BCN-348 across the *c*-axis; (d) the assembly of face-sharing cuboctahedron stacking; and (e) the spatial distribution of octahedral cages around the cuboctahedral cavity.

Next, we performed topological analysis of the structure using ToposPro 5.3.3.5 software^31^ and following literature recommendations to deconstruct the linker into more-regular triangular and square shapes, which revealed the formation of a 3,4,8-c eps net.^32^ To the best of our knowledge, this topology had never previously been observed in Zr- or RE-based MOFs, and there is just one reported example of a structure (a Ni-based MOF) exhibiting this net.^8^ Also, when we performed a topological analysis without splitting the linker but instead, treating it as an octagonal node, the connectivities of this node and of the Zr-oxocluster were halved, resulting in a 4,4-c nbo-b net. This finding proves that the eps net is a nbo-b-related net and therefore, corroborates that BCN-348 is the first Zr-MOF based on this related net.^9,33^

Seeking to understand the formation of the aforementioned eps net, we did a reticular survey.^1*b*^ According to the RCSR database, combining an octagonal-shape linker with a Zr-cluster, which could be 12-c, 8-c, 6-c or 4-c (in the case of the most-regular ones), would lead to the formation of kce (3,4,12-c), eps (3,4,8-c), cye (6,8-c), cze (3,4,6-c), or cyt (4,8-c) nets, respectively. Among these, we initially excluded both the cye and the cyt nets, due to the mismatch between the proximity of two neighbouring Zr-clusters in these nets as well as to the length of the TBCPB linker that we had used. Regarding the kce net, this topology had already been reported as resulting from the combination of the TBCPB linker with a 12-c nonanuclear RE-cluster that exhibits a hexagonal prism conformation (Fig. 1). However, the strong Zr–O bonds always favour the formation of the hexanuclear Zr-oxocluster, enabling defaults in its connectivity,^11–18,20–28^ rather than being disordered in its 12-c hexagonal prism conformation.^19,29^ We hypothesise that together, these features favoured the formation of the eps net rather than either the kce net, which would have involved the formation of a disordered 12-c hexagonal prismatic Zr-cluster, or the cze net, which is less connected than eps (Fig. 1).

Next, we confirmed the phase purity of the bulk BCN-348 sample by powder X-ray diffraction (PXRD): the experimental results matched the simulated ones that we had obtained by SCXRD (Fig. S1, ESI†). We then studied the porosity of BCN-348 by running N_2_-sorption experiments at 77 K. BCN-348 exhibits an apparent Brunet–Emmett–Teller (BET) area of 2564 m^2^ g^−1^ in an isotherm between type-Ib and type-IV (Fig. S3–S5, ESI†). The total pore volume was found to be 1.14 cm^3^ g^−1^ at *P*/*P*_0_ = 0.95. The pore-size distribution, based on DFT models, revealed the presence of mesopores of 27.3 Å and 31.7 Å (Fig. S6, ESI†). Moreover, BCN-348 was thermally stable up to 508 °C (Fig. S7, ESI†), and it maintained its phase crystallinity when incubated in water for 1 h, 12 h and 24 h (Fig. S2, ESI†). The fact that BCN-348 combines mesoporosity with thermal and hydrolytic stability illustrates the feasibility of using high-connected clusters and linkers to assemble robust porous MOFs.

Among the most promising recent applications for Zr-MOFs is their use as hydrolytic catalysts for the detoxification of highly toxic organophosphate compounds—namely pesticides, and nerve agents and their simulants.^34^ These detoxification processes imply the activation of the P–X (X= O, S, F) bond by zirconium Lewis acidic and hydroxide basic sites at the oxohydroxide Zr-cluster. Consequently, pore size and polarity, framework connectivity, and stability together define the access of water molecules and toxic substrate molecules to the catalytically active metal cluster. To date, most of these Zr-MOF-assisted catalytic processes have been done using simulants with ester P–O bonds, together with corrosive and toxic basic buffers (*N*-ethylmorpholine). Importantly, the toxicity of common nerve agents such as Soman (GD), Sarin (GB) and Cyclosarin (GF) is determined by labile P–F bonds. Consequently, there is pronounced interest in the development of new detoxification materials that could hydrolyse the P–F bonds of hazardous chemicals in unbuffered aqueous solutions.

Seeking to exploit the accessible pore structure and hydrolytic stability of BCN-348, we tested its catalytic activity in the removal of the simulant diisopropylfluorophosphate (DIFP), which contains a P–F bond. We ran the hydrolysis in an aqueous unbuffered dispersion of BCN-348 using a DIFP/MOF ratio of 1.5 : 1. The removal of DIFP from the solution was first followed by GC. The half-life of detoxification is 72.2 min with quantitative removal of the toxic simulant after 24 hours (Fig. 3). To have a deep insight into the detoxication mechanism, we performed additional ^31^P NMR and ^1^H NMR analysis in D_2_O. The results confirmed that DIFP is removed from the supernatant solution after 24 hours, with diisopropylphosphate (DIP) appearing as the main hydrolytic product (72.8%). However, there was still a fraction of 27.2% of unreacted DIFP molecules, which were mostly adsorbed by BCN-348 (Table S2 and Fig. S8–S10, ESI†). These results confirm the highly accessible porous structure and exposed nature of the Zr metal sites in the metal clusters.

**Fig. 3:**
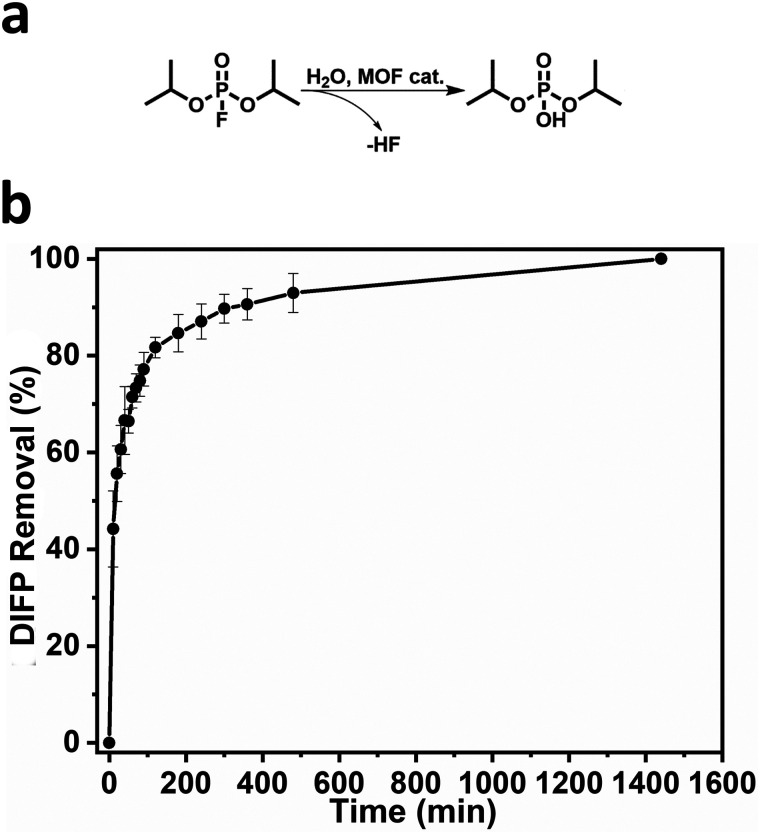
(a) Hydrolysis of DIFP catalysed by BCN-348 (denoted as “MOF cat.”). (b) DIFP removal plotted against time.

In conclusion, we have reported the design, synthesis and functional validation of BCN-348, a new mesoporous high-connected Zr-MOF, which we assembled by connecting an 8-c cubic Zr-oxocluster through an 8-c organic linker. Topologically, BCN-348 exhibits a minimal edge-transitive eps net, unlike its RE analogue, which exhibits the minimal edge-transitive kce net. Moreover, BCN-348 combines mesoporosity with the presence of Lewis-acid open metal sites and hydrolytic stability, permitting its use as a catalyst for the hydrolytic degradation of toxic chemicals without any assistance from a buffer, which we confirmed on DIFP, a nerve-agent simulant. Our study augments the collection of reticular Zr-MOFs and highlights the endless possibilities of assembling regular, high-connected, molecular building blocks in symmetric reticular materials, thus further paving the way to new high-connected frameworks.

This work has received funding from the European Union's Horizon 2020 research and innovation programme, under grant agreement No 101019003, the Catalan AGAUR (project 2021 SGR 00458), the CERCA Programme/Generalitat de Catalunya, and the Spanish MCIN/AEI/10.13039/501100011033 (Project PID2020-113608RB-I00). ICN2 is supported by the Severo Ochoa Centres of Excellence programme, Grant CEX2021-001214-S, funded by MCIN/AEI/10.13039.501100011033.

## Conflicts of interest

There are no conflicts to declare.

## Supplementary Material

CC-059-D3CC01831H-s001

CC-059-D3CC01831H-s002
