# Supplementary material for: A mesoporous Zr-based metal–organic framework driven by the assembly of an octatopic linker
Source: Chem Commun (Camb). 2023 May 25;59(50):7803–6. doi: 10.1039/d3cc01831h (PMC10281490; doi:10.1039/d3cc01831h)
Supplement: CC-059-D3CC01831H-s001 [file CC-059-D3CC01831H-s001.pdf]

Electronic Supplementary Material (ESI) for ChemComm.
This journal is © The Royal Society of Chemistry 2023

Supporting information

A mesoporous Zr-based metal-organic framework driven by the assembly of an octatopic linker

Borja Ortín-Rubio,[a,b] Cristina Perona-Bermejo,[c] José A. Suárez del Pino,[a,b] Francisco J. Carmona,[c] Felipe Gándara,[d] Jorge A. R. Navarro,[c] Judith Juanhuix,[e] Inhar Imaz,*[a] and Daniel Maspoch *[a,b,f]

[a.] *Catalan Institute of Nanoscience and Nanotechnology (ICN2), CSIC and The Barcelona Institute of Science and Technology, Campus UAB, Bellaterra, 08193 Barcelona, Spain*
[b.] *Departament de Química, Facultat de Ciències, Universitat Autònoma de Barcelona, 08193 Bellaterra, Spain*
[c.] *Departamento de Química Inorgánica, Universidad de Granada, Av. Fuentenueva S/N, 18071 Granada, Spain*
[d.] *Materials Science Institute of Madrid (ICMM), Consejo Superior de Investigaciones Científicas (CSIC), Calle Sor Juana Inés de la Cruz, 3, 28049 Madrid, Spain*
[e.] *ALBA Synchrotron, 08290 Cerdanyola del Vallès, Barcelona, Spain*
[f.] *ICREA, Pg. Lluís Companys 23, 08010 Barcelona, Spain*

Table of contents

S1. Materials, methods and characterization 3

S1.1. Materials 3

S1.2. Methods 3

S1.3. Characterization 3

S2. Characterization of BCN-348 5

S2.1. Single-crystal X-ray diffraction 5

S2.2. Powder X-Ray Diffraction 6

S2.3. N_2 sorption 8

S2.4. Thermogravimetric Analysis 12

S3. Heterogeneous degradation of simulant diisopropylfluorophosphate (DIFP) in unbuffered solution 13

S3.1. Gas Chromatographic (GC) studies 13

S3.2. ^{1}H and ^{31}P-NMR studies 13

S4. References 17

S1. Materials, methods and characterization

S1.1. Materials

Zirconium oxychloride octahydrate ($ZrOCl_2·8H_2O$), potassium carbonate (K_2CO_3), potassium iodide (KI), sodium hydroxide (NaOH) and bis(triphenylphosphine)palladium(II) dichloride ($PdCl_2[P(Ph)_3]_2$) were purchased from Sigma-Aldrich Co. *N,N*-dimethylformamide (DMF), acetonitrile (MeCN), tetrahydrofuran (THF), hexane, ethylacetate (AcOEt), methanol (MeOH), hydrochloric acid (HCl) and sulfuric acid (H_2SO_4) were obtained from Fisher Chemical. Trifluoroacetic acid (TFA) was obtained from TCI. 3,5-dibromophenol, 4-carboxyphenylboronic acid and 1,2,4,5-tetrakis(bromomethyl)benzene were purchased from BLDpharm. All the reagents and solvents were used without further purification unless otherwise specified. Deionized water was obtained with a Milli-Q® system (18.2 MΩ·cm).

S1.2. Methods

Synthesis of 1,2,4,5-tetrakis[3,5-bis(4-carboxyphenyl)phenoxymethyl]benzene (H_8TBCPB)

1,2,4,5-tetrakis[3,5-bis(4-carboxyphenyl)phenoxymethyl]benzene was synthesized according to a previous reported procedure.[1]

Synthesis of BCN-348

A solution of $ZrOCl_2·8H_2O$ (26 mg, 0.08 mmol) and H_8TBCPB (58 mg, 0.04 mmol) in DMF (2 mL) and trifluoroacetic acid (0.6 mL) was prepared in a 23 mL scintillation vial. Then, the sealed vial was placed into a preheated oven at 120 ºC for 5 days. After this period, colourless cubic crystals suitable for single-crystal X-ray diffraction (SCXRD) were collected by filtration and washed three times by incubating them with 20 mL of fresh DMF for 12 h. Afterwards, solvent exchange with acetone was performed by incubating the crystals three times with 20 mL of acetone for 12 h, and the resulting BCN-348 crystals were dried at room temperature.

S1.3. Characterization

Single-Crystal X-Ray Diffraction (SCXRD) data for BCN-348 were collected at 100 K at XALOC beamline at ALBA synchrotron (0.82656 Å).[2] Data were indexed, integrated and scaled using the XDS program.[3] Absorption correction was not applied. The structures were solved by direct methods and subsequently refined by correction of F2 against all reflections, using SHELXT2018 within Olex2 package.[4-6] All non-hydrogen atoms were refined with anisotropic thermal parameters by full-matrix least-squares calculations on F2 using the program SHELXL2018.[5] We treated the presence of solvent molecules in the cavities of all structures running solvent mask using Olex2 solvent mask.[6] The structure was solved using two space groups: Fm-3m and Fm-3. Although the correct symmetry corresponding to the structure is Fm-3m, we could not model the disorder of the central benzene ring of the linker (which could not be clearly defined using the maps) using this space group. For this reason, we decided to solve the crystal structure using F-3m group, which allowed us to model this benzene ring applying some restraints, such as DFIX, DELU and SIMU. The hydrogen atoms were calculated in their expected positions with the HFIX instruction of SHELXL2018, and refined as riding atoms with Uiso(H) = 1.5 Ueq(C).

Powder X-Ray Diffraction (PXRD) diagrams were collected on a Panalytical X'pert mpd diffractometer with monochromatic Cu-Kα radiation (λ_{Cu} = 1.5406 Å).

Proton Nuclear Magnetic Resonance (^{1}H NMR) spectra were acquired in Bruker Avance DPX of 250 MHz, 360 MHz NMR spectrometers at "Servei de Ressonància Magnètica Nuclear" from Autonomous University of Barcelona (UAB).

Proton and Phosphorus Nuclear Magnetic Resonance (^{1}H NMR/^{31}P NMR) spectra (for the catalytic experiments) were obtained in a BRUKER Nanobay Avance III HD High Definition 400 MHz (2-channel) NMR spectrometer.

Thermogravimetric analyses (TGA) were performed in a Pyris TGA8000, heating the sample from 25 ºC to 800 ºC at 1 ºC/min under N_2 atmosphere.

N_2 sorption isotherms were collected at 77K (N_2) using an ASAP 2460 HD (Micromeritics). Temperature was controlled by using a liquid nitrogen bath. Prior to the measurements, the samples were activated by solvent exchange with acetone, 3 times per day during 3 days. The specific surface area was obtained using the Brunauer-Emmett-Teller (BET) adsorption method in the range of 0.001 – 0.02 P/P_0 (BETSI software).[7] Total pore volume (V_t) was calculated at P/P_0 = 0.95. Pore size distribution was estimated using a density functional theory (DFT) model (HS-2D-NLDFT Carbon Cylindrical Mesopores @ N_2 77K) implemented in the Microactive 4.06 software with a regularization factor of 0.03160.

Gas Chromatography analyses (GC) were carried out in an Agilent 8860 GC chromatograph with a FID dectector and a 16 port autosampler. A HP-5 column of 50 m length, 0.320 mm diameter and 1.05 µm thickness was used, which allowed working from -60 ºC to 325 ºC.

S2. Characterization of BCN-348

S2.1. Single-crystal X-ray diffraction

**S1. Crystal data and structure refinement for BCN-348**	
Identification code	CCDC-2255518
Empirical formula	$C_{369}H_{702}N_{94}O_{130}Zr_6$
Formula weight	9083.52
Temperature/K	100.0
Crystal system	cubic
Space group	Fm-3
a/Å	59.677(2)
b/Å	59.677(2)
c/Å	59.677(2)
α/°	90
β/°	90
γ/°	90
Volume/Å^3	212530(21)
Z	24
ρ_{calc}g/cm^3	1.703
μ/mm^{-1}	0.427
F(000)	116496.0
Crystal size/mm^3	0.07 × 0.07 × 0.07
Radiation	synchrotron (λ = 0.82656)
2Θ range for data collection/°	1.374 to 49.596
Index ranges	-60 ≤ h ≤ 60, -60 ≤ k ≤ 60, -60 ≤ l ≤ 60
Reflections collected	297552
Independent reflections	10067 [R_{int} = 0.1572, R_{sigma} = 0.0390]
Data/restraints/parameters	10067/104/286
Goodness-of-fit on F^2	1.186
Final R indexes [I>=2σ (I)]	R_1 = 0.1167, wR_2 = 0.3015
Final R indexes [all data]	R_1 = 0.1450, wR_2 = 0.3217
Largest diff. peak/hole / e Å^{-3}	0.50/-2.15

S2.2. Powder X-Ray Diffraction

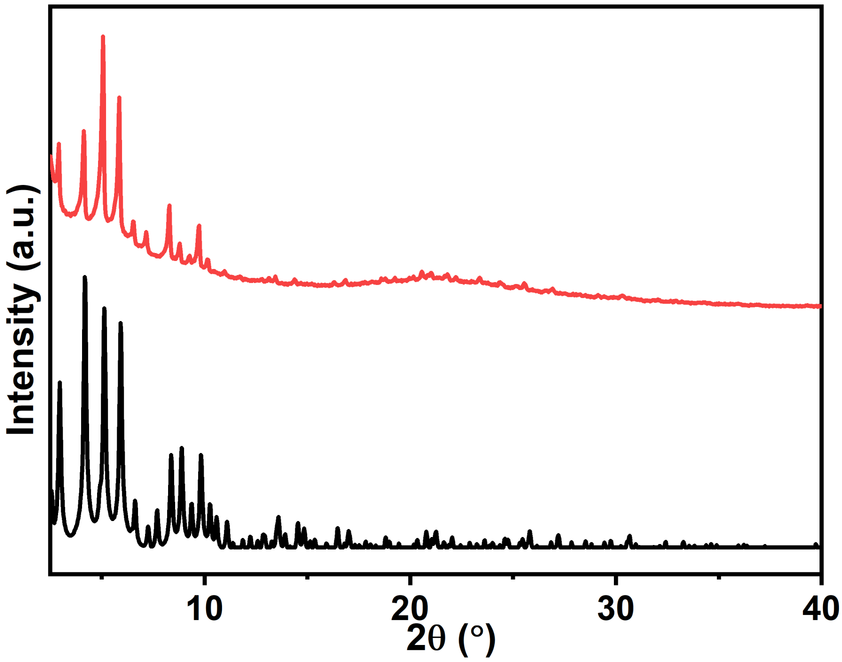

Figure S1. PXRD pattern of calculated BCN-348 (black) and pristine BCN-348 (red).

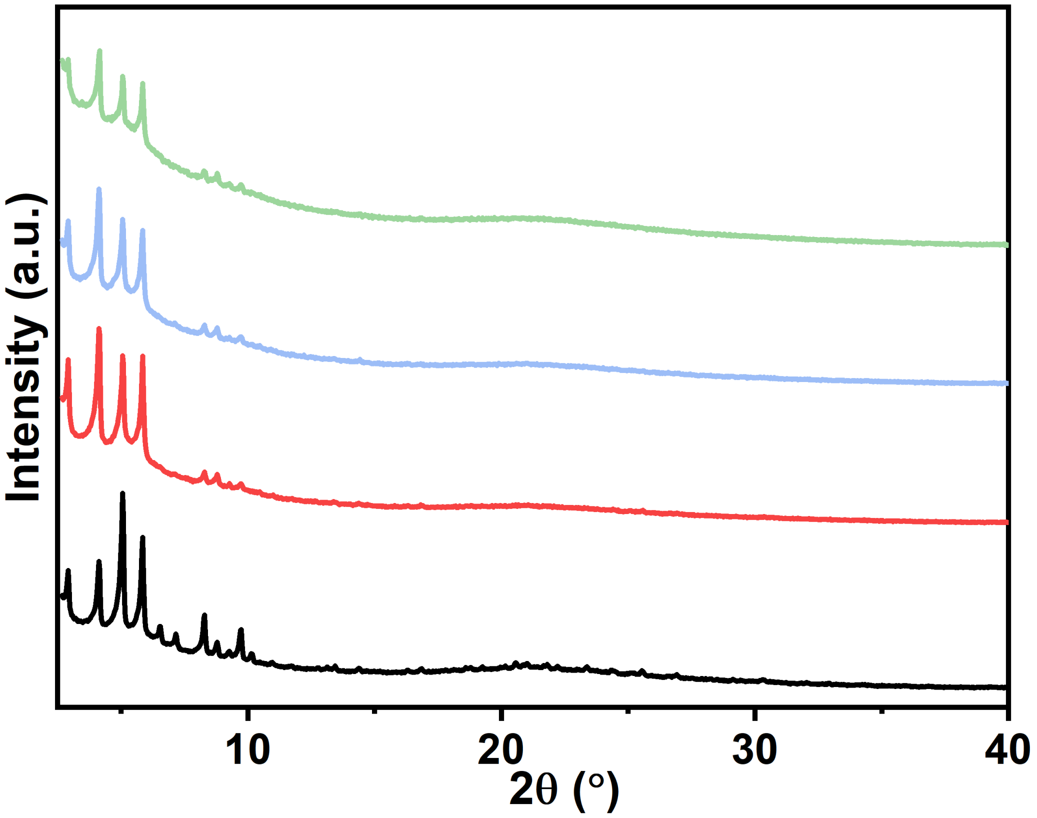

Figure S2. PXRD patterns of pristine BCN-348 (black) and after its incubation in water for 1 h (red), 12 h (blue) and 24 h (green).

S2.3. N_2 sorption

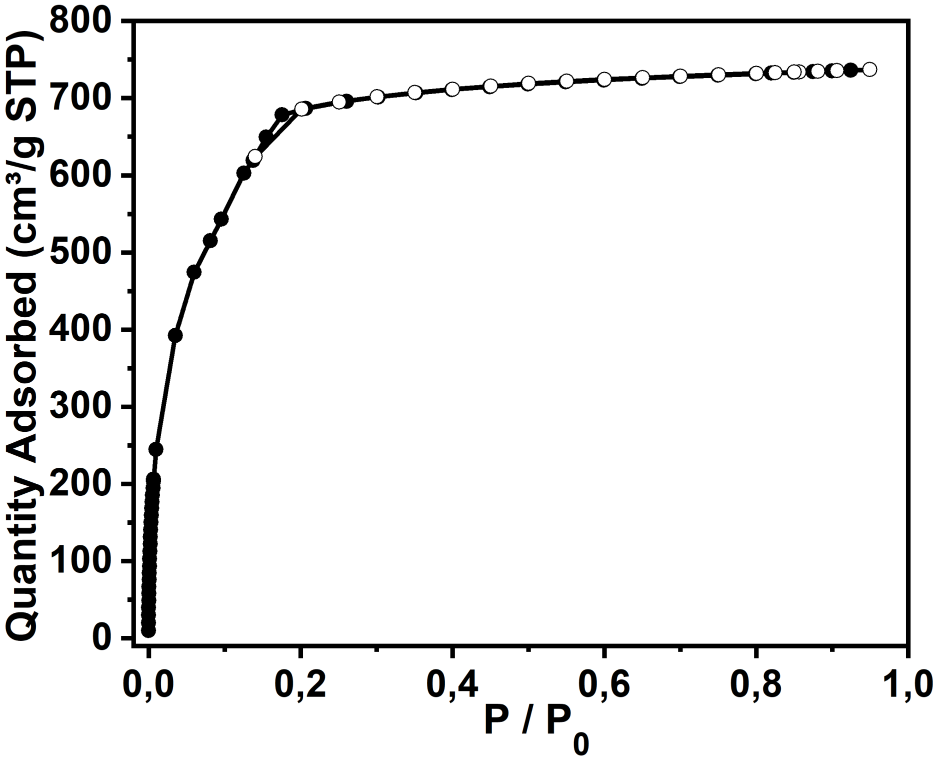

Figure S3. N_2 sorption (filled symbols) and desorption (empty symbols) isotherms at 77 K of BCN-348.

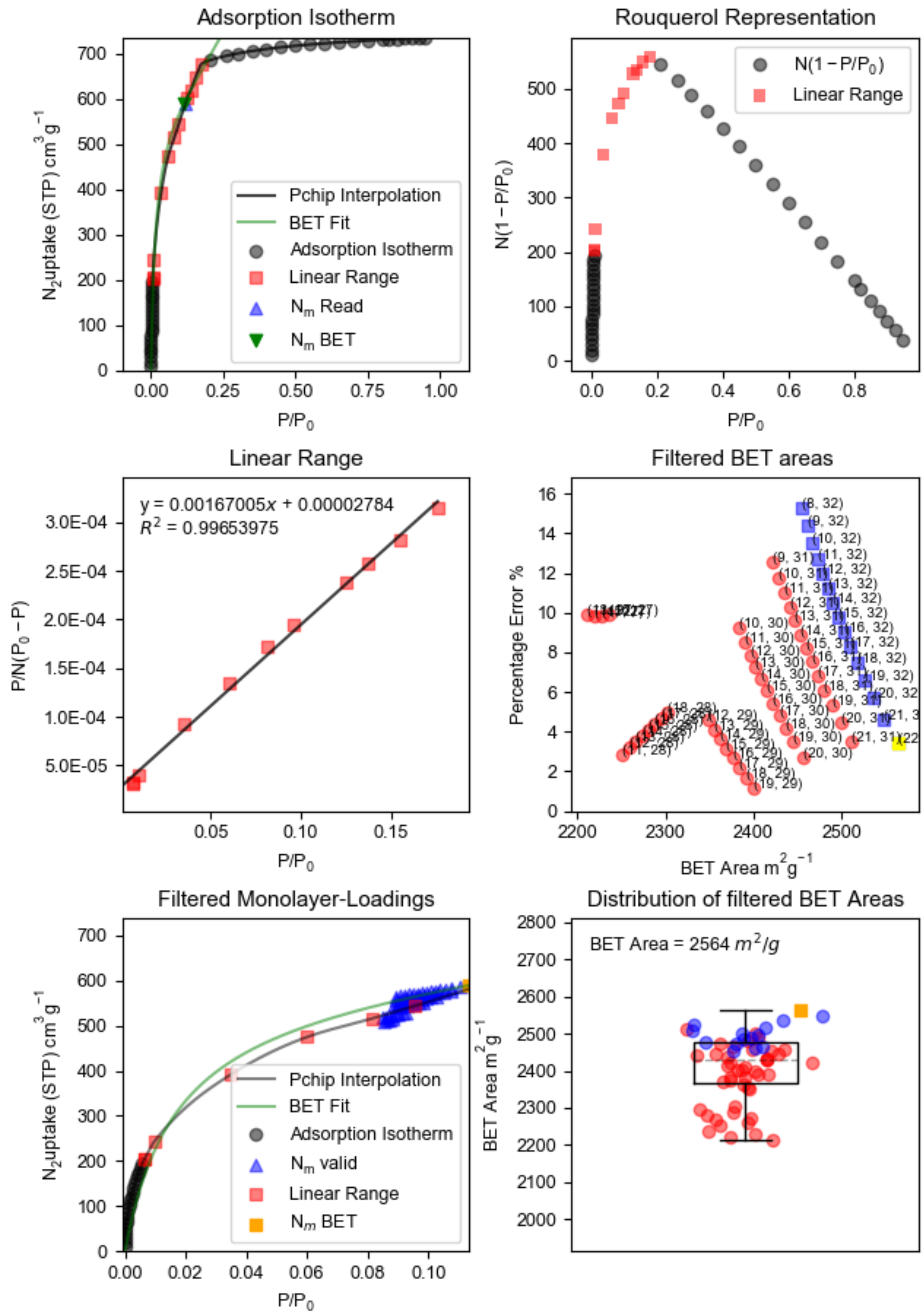

Figure S4. BETSI analysis for BCN-348.

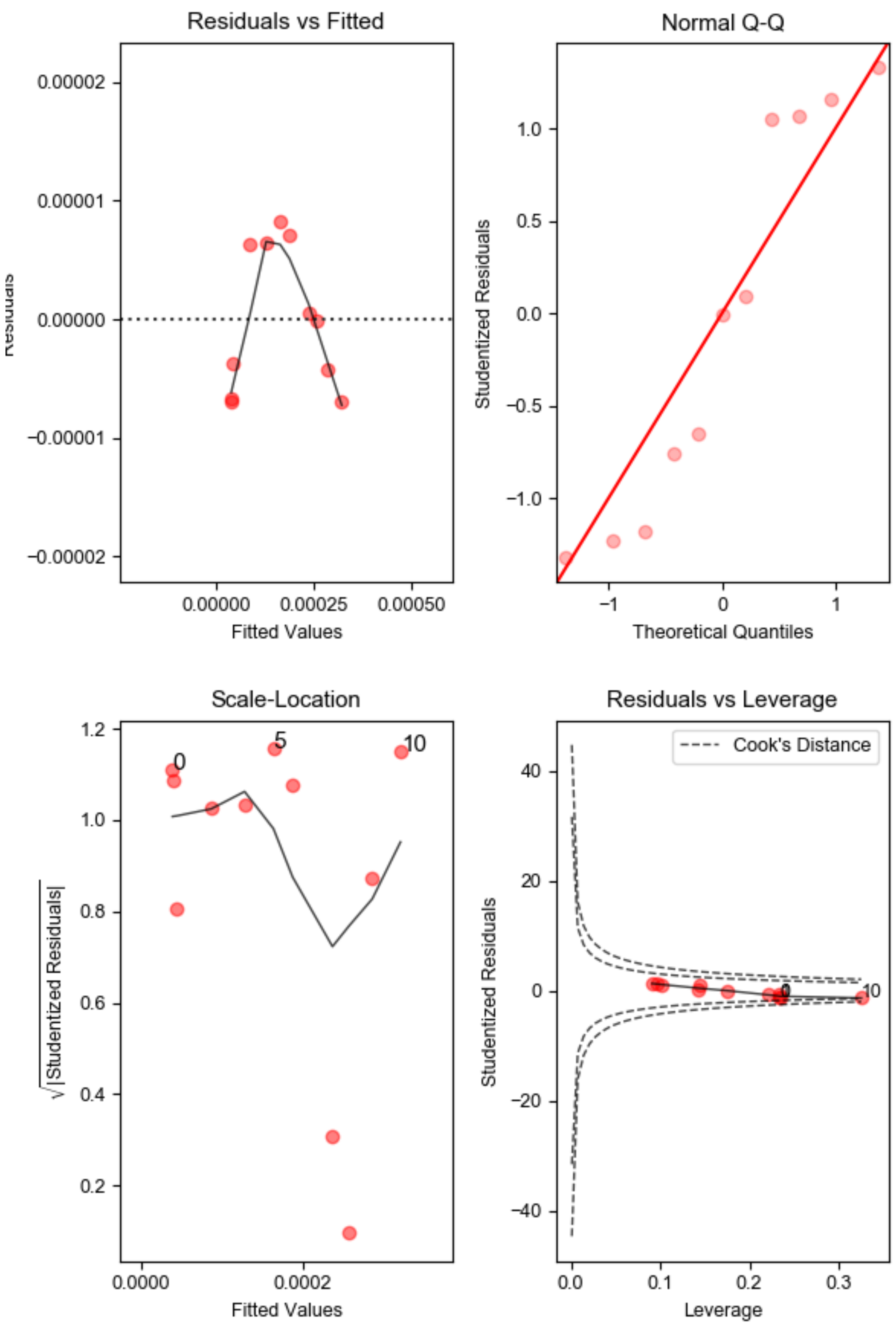

Figure S5. BETSI regression diagnostics for BCN-348.

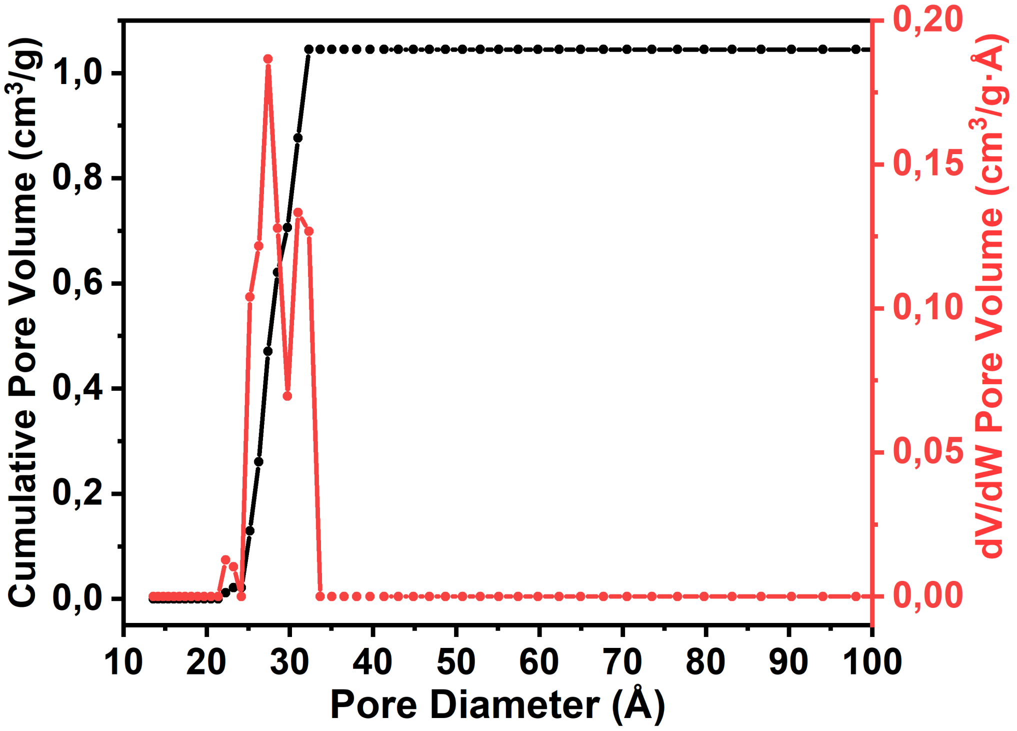

Figure S6. Pore size distribution estimated by DFT for BCN-348.

S2.4. Thermogravimetric Analysis

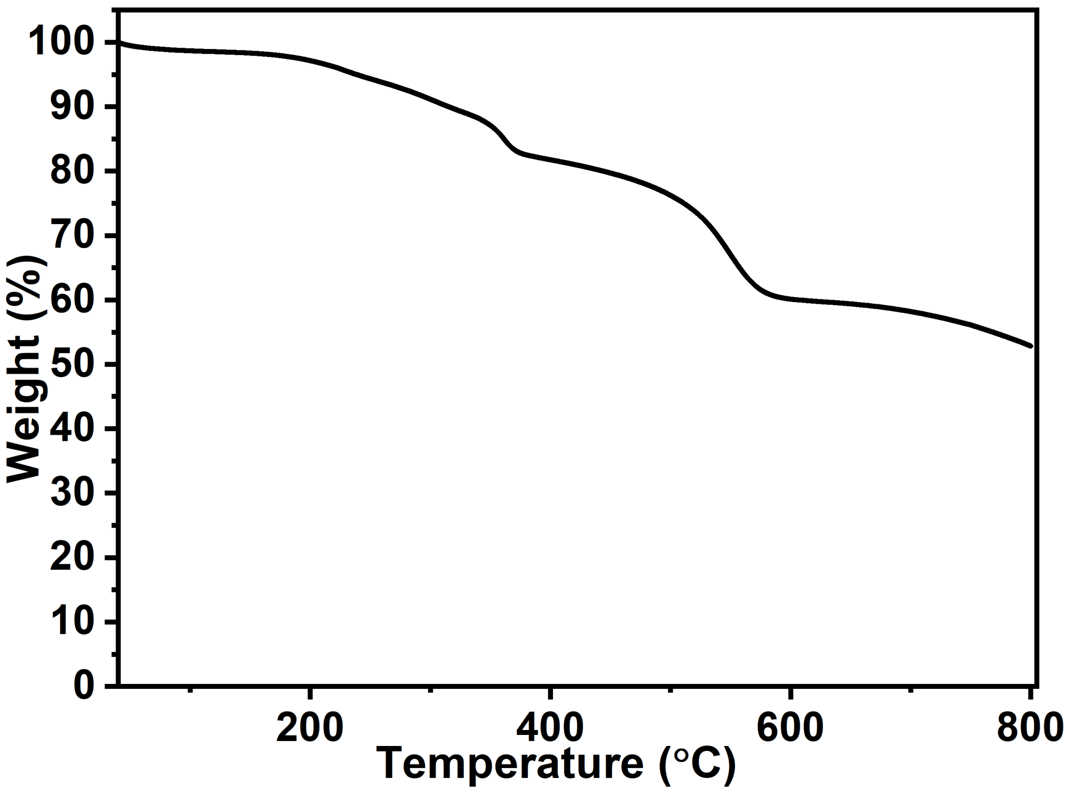

Figure S7. TGA analysis of activated BCN-348. The weight loss from ~ 200 to 450 ºC can be attributed to the detachment of partial formates (coming from the degradation of DMF) or trifluoroacetates in the structure, as typically observed in Zr-MOFs.[8,9]

S3. Heterogeneous degradation of simulant diisopropylfluorophosphate (DIFP) in unbuffered solution

S3.1. Gas Chromatographic (GC) studies

The removal of DIFP was studied employing 12 mg of BCN-348 (2.5 µmol) suspended in 0.6 mL of H_2O. Afterwards, 1.5 µL of *N,N*-dimethylacetamide (DMA), used as internal reference, and 1.5 µL of DIFP (3.8 µmol) were added to the suspension (DIFP/MOF ratio of 1.5:1). The evolution of the concentration of DIFP in the supernatant solution was followed at room temperature by means of GC.

S3.2. ^{1}H and ^{31}P NMR studies

The degradation of DIFP nerve gas simulant into diisopropylphosphate (DIP) by BCN-348 was followed by ^{1}H NMR and ^{31}P NMR spectroscopy. Specifically, 12 mg of BCN-348 were suspended in a mixture containing 0.6 mL of D_2O, 0.7 µL of dimethylphosphate (DMP, used as internal reference) and 1.5 µL of DIFP (DIFP/MOF ratio of 1.5:1). The suspension was stirred during 24 hours at room temperature and the concentration of DIPF and DIP in the supernatant solution and in the adsorbate phase were quantified by NMR.

After 24 hours, the solid was separated from the supernatant by centrifugation (10000 rpm, 5 min). Afterwards, the solid was suspended in $CDCl_3$ for 5 hours at room temperature to extract DIFP molecules adsorbed inside the cavities of BCN-348 and the concentration of DIFP and its degradation product DIP were determined by ^{1}H-NMR.

Table S2. Percentage distribution of unreacted and hydrolyzed DIFP in the supernatant and adsorbate phase after 24 hours of incubation with BCN-348 as determined from ^{1}H NMR in D_2O (0.6 mL) and after extraction with $CDCl_3$ (0.6 mL), respectively.

Hydrolyzed DIFP (DIP) (%)		Non-hydrolyzed DIFP (%)	
**Supernatant**	**Adsorbed[a]**	**Supernatant**	**Adsorbed**
12.8	60	2.2	25

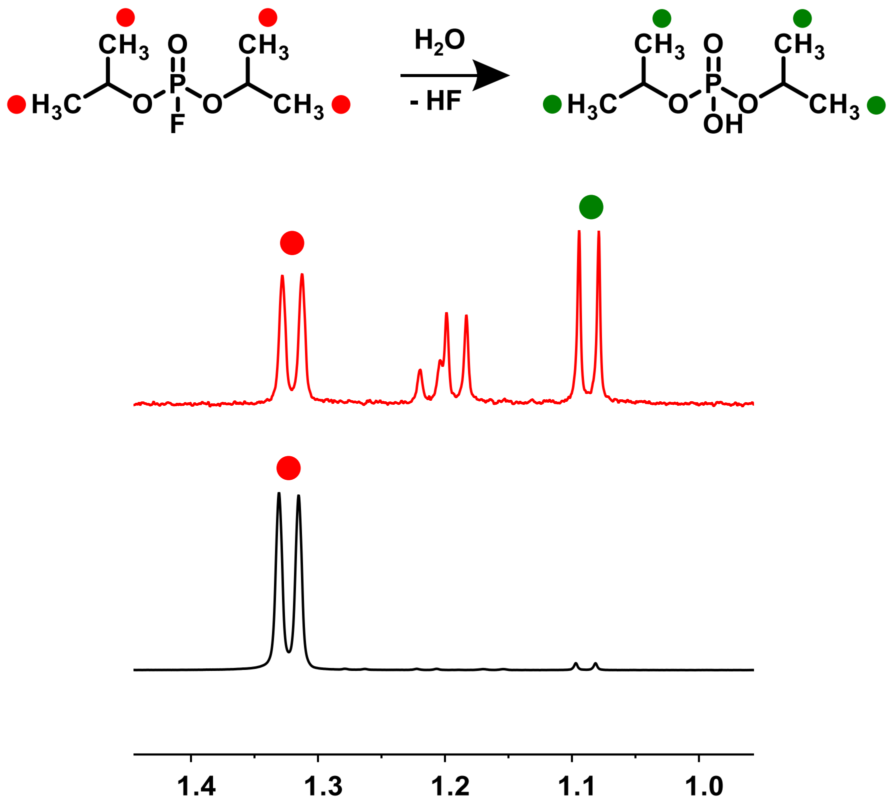

Figure S8. ^{1}H NMR spectra of supernatant after 24 h (top) and 0 h (bottom) suspended of BCN-348 with DIFP in unbuffered solution.

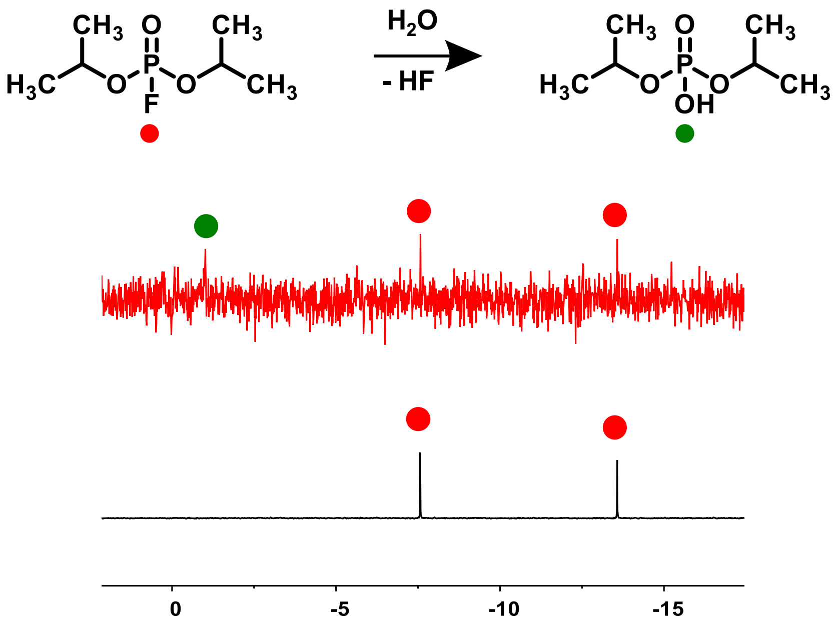

Figure S9. ^{31}P NMR spectra of supernatant after 24 h (top) and 0 h (bottom) suspended of BCN-348 with DIFP in unbuffered solution.

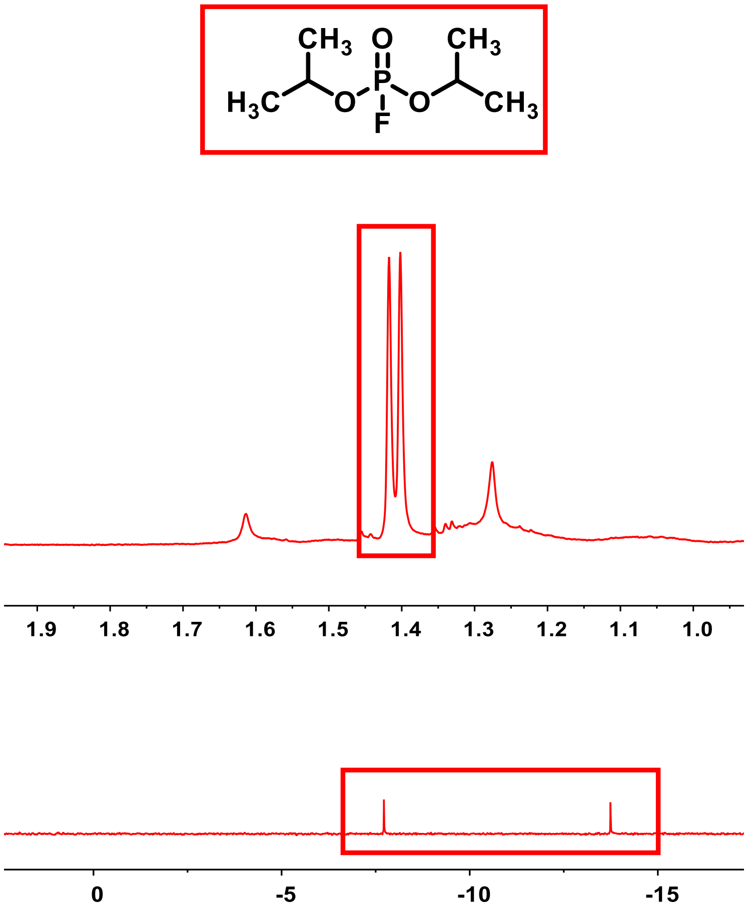

Figure S10. ^{1}H NMR (top) and ^{31}P NMR (bottom) spectra of extracted solution from BCN-348 solid previously suspended during 24 hours with DIFP in unbuffered solution. Extraction solvent: $CDCl_3$.

S4. References

1 Z. Chen, Z. Thiam, A. Shkurenko, L. J. Weselinski, K. Adil, H. Jiang, D. Alezi, A. H. Assen, M. O'Keeffe and M. Eddaoudi, *J. Am. Chem. Soc.*, 2019, **141**, 20480–20489.

2 J. Juanhuix, F. Gil-Ortiz, G. Cuní, C. Colldelram, J. Nicolás, J. Lidón, E. Boter, C. Ruget, S. Ferrer and J. Benach, *J. Synchrotron Radiat.*, 2014, **21**, 679–689.

3 W. Kabsch, *Acta Cryst. D*, 2010, **66**, 133–144.

4 G. M. Sheldrick and IUCr, *Acta Cryst. A*, 2015, **71**, 3–8.

5 G. M. Sheldrick and IUCr, *Acta Cryst. C*, 2015, **71**, 3–8.

6 O. V. Dolomanov, L. J. Bourhis, R. J. Gildea, J. A. K. Howard and H. Puschmann, *J. Appl. Cryst.*, 2009, **42**, 339–341.

7 J. W. M. Osterrieth, J. Rampersad, D. Madden, N. Rampal, L. Skoric, B. Connolly, M. D. Allendorf, V. Stavila, J. L. Snider, R. Ameloot, J. Marreiros, C. Ania, D. Azevedo, E. Vilarrasa-Garcia, B. F. Santos, X. H. Bu, Z. Chang, H. Bunzen, N. R. Champness, S. L. Griffin, B. Chen, R. B. Lin, B. Coasne, S. Cohen, J. C. Moreton, Y. J. Colón, L. Chen, R. Clowes, F. X. Coudert, Y. Cui, B. Hou, D. M. D'Alessandro, P. W. Doheny, M. Dincă, C. Sun, C. Doonan, M. T. Huxley, J. D. Evans, P. Falcaro, R. Ricco, O. Farha, K. B. Idrees, T. Islamoglu, P. Feng, H. Yang, R. S. Forgan, D. Bara, S. Furukawa, E. Sanchez, J. Gascon, S. Telalović, S. K. Ghosh, S. Mukherjee, M. R. Hill, M. M. Sadiq, P. Horcajada, P. Salcedo-Abraira, K. Kaneko, R. Kukobat, J. Kenvin, S. Keskin, S. Kitagawa, K. ichi Otake, R. P. Lively, S. J. A. DeWitt, P. Llewellyn, B. v. Lotsch, S. T. Emmerling, A. M. Pütz, C. Martí-Gastaldo, N. M. Padial, J. García-Martínez, N. Linares, D. Maspoch, J. A. Suárez del Pino, P. Moghadam, R. Oktavian, R. E. Morris, P. S. Wheatley, J. Navarro, C. Petit, D. Danaci, M. J. Rosseinsky, A. P. Katsoulidis, M. Schröder, X. Han, S. Yang, C. Serre, G. Mouchaham, D. S. Sholl, R. Thyagarajan, D. Siderius, R. Q. Snurr, R. B. Goncalves, S. Telfer, S. J. Lee, V. P. Ting, J. L. Rowlandson, T. Uemura, T. Iiyuka, M. A. van der Veen, D. Rega, V. van Speybroeck, S. M. J. Rogge, A. Lamaire, K. S. Walton, L. W. Bingel, S. Wuttke, J. Andreo, O. Yaghi, B. Zhang, C. T. Yavuz, T. S. Nguyen, F. Zamora, C. Montoro, H. Zhou, A. Kirchon and D. Fairen-Jimenez, *Adv. Mat.*, 2022, **34**, 2201502.

8 Y. Zhang, X. Zhang, J. Lyu, K. I. Otake, X. Wang, L. R. Redfern, C. D. Malliakas, Z. Li, T. Islamoglu, B. Wang and O. K. Farha, *J. Am. Chem. Soc.*, 2018, **140**, 11179–11183.

9 H. A. Bicalho, F. Saraci, J. de J. Velzquez-Garcia, H. M. Titi and A. J. Howarth, *Chem. Commun.*, 2022, **58**, 10925-10928.
